# Presumed consent for organ preservation in uncontrolled donation after cardiac death in the United States: a public policy with serious consequences

**DOI:** 10.1186/1747-5341-4-15

**Published:** 2009-09-22

**Authors:** Joseph L Verheijde, Mohamed Y Rady, Joan McGregor

**Affiliations:** 1 Bioethics, Policy and Law Program, School of Life Sciences, Center for Biology and Society, Arizona State University, 300 East University Drive, Tempe, Arizona 85287, USA; 2 Department of Biomedical Ethics, College of Medicine, Mayo Clinic, 5777 East Mayo Boulevard Phoenix, AZ 85054, USA; 3 Department of Physical Medicine and Rehabilitation, Mayo Clinic Hospital, 5777 East Mayo Boulevard, Phoenix, Arizona, 85054, USA; 4 Department of Critical Care Medicine, Mayo Clinic Hospital, 5777 East Mayo Boulevard, Phoenix, Arizona, 85054, USA; 5 Department of Philosophy, Arizona State University, 300 East University Drive, Tempe, Arizona 85287, USA

## Abstract

Organ donation after cessation of circulation and respiration, both controlled and uncontrolled, has been proposed by the Institute of Medicine as a way to increase opportunities for organ procurement. Despite claims to the contrary, both forms of controlled and uncontrolled donation after cardiac death raise significant ethical and legal issues. Identified causes for concern include absence of agreement on criteria for the declaration of death, nonexistence of universal guidelines for duration before stopping resuscitation efforts and techniques, and assumption of presumed intent to donate for the purpose of initiating temporary organ-preservation interventions when no expressed consent to donate is present. From a legal point of view, not having scientifically valid criteria of cessation of circulation and respiration for declaring death could lead to a conclusion that organ procurement itself is the proximate cause of death. Although the revised Uniform Anatomical Gift Act of 2006 provides broad immunity to those involved in organ-procurement activities, courts have yet to provide an opinion on whether persons can be held liable for injuries arising from the determination of death itself. Preserving organs in uncontrolled donation after cardiac death requires the administration of life-support systems such as extracorporeal membrane oxygenation. These life-support systems can lead to return of signs of life that, in turn, have to be deliberately suppressed by the administration of pharmacological agents. Finally, allowing temporary organ-preservation interventions without expressed consent is inherently a violation of the principle of respect for a person's autonomy. Proponents of organ donation from uncontrolled donation after cardiac death, on the other hand, claim that these nonconsensual interventions enhance respect for autonomy by allowing people, through surrogate decision making, to execute their right to donate organs. However, the lack of transparency and the absence of protection of individual autonomy, for the sake of maximizing procurement opportunities, have placed the current organ-donation system of opting-in in great jeopardy. Equally as important, current policies enabling and enhancing organ procurement practices, pose challenges to the constitutional rights of individuals in a pluralistic society as these policies are founded on flawed medical standards for declaring death.

## Introduction

Since the first kidney transplant in 1954, transplantation has been an area of medicine of utmost complexity and has often been a polarizing one as well [1]. Nevertheless, as of April 17, 2009, the United Network for Organ Sharing and Organ Procurement and Transplantation Network reported that 101,761 persons are waiting for organ transplantation, and this number continues to grow [2]. To increase the number of organs available for transplantation, major initiatives have been implemented, and new strategies are being developed. Before 1968, organs were procured exclusively from donors with cardiorespiratory failure, ie, non-heart-beating organ donation. The Harvard Ad Hoc Committee subsequently defined irreversible coma as "brain death," equating "brain death" with human death and, in so doing, opened the way to procure organs from heart-beating donors [3]. Because of the increasing number of patients in need of organs for transplantation and the subsequent growing disparity between supply and demand for transplantable organs [4], the US Department of Health and Human Services asked the Institute of Medicine (IOM) to form a committee with the charge to conduct a review of proposals and efforts to increase organ donation. This request was made in response to the legislative requirements laid down in the Organ Donation and Recovery Improvement Act of 2004. The 2006 IOM Committee was charged with the development of strategies to improve organ-donation rates, taking into consideration ethical implications, possible impact on public perceptions, cost-effectiveness, feasibility, and practicality of implementing such proposals [5]. The Committee proposed to expand the pool of potential donors, postulating that out of the roughly 335,000 annual deaths by cardiac arrest, at least 22,000 individuals would meet the criteria for uncontrolled donation after cardiac death (uDCD). In controlled DCD, circulatory arrest follows consented removal of mechanical ventilation and hemodynamic support from a critically ill patient, who cannot be declared "brain dead" and meets specific cardiocirculatory criteria for death after life support is taken away [6]. uDCD involves procuring organs from patients either in whom unsuccessful resuscitation efforts are terminated in the field or the emergency department or after they being brought to the hospital dead on arrival [7]. uDCD raises ethical and legal concerns similar to those in controlled DCD, some of which have been previously addressed, including lack of scientific validation of (1) the current circulatory criteria for determining death and (2) the standard medical tests used to confirm that uniform criteria of death are fulfilled [8-10]. Other ethical and legal concerns specific to uDCD include (1) the absence of universal guidelines that determine duration before resuscitation efforts are stopped; (2) presumed authority in the absence of first-person consent to initiate temporary organ-preservation (TOP) techniques; (3) the administration of advanced, highly invasive preservation techniques; and (4) the potential for conflicts of obligation, loyalty, and interests among the stakeholders. These conflicts revolve around the responsibility to secure the best interest of the patient (within the context of the traditional physician-patient relationship) versus ensuring the health of the donor's organs for an anticipated recipient (within the context of the organ harvester-donor relationship) before the patient has become a donor. All of these concerns must be addressed and resolved to avoid any further erosion of public's trust in the integrity of medical practice.

## The criteria of circulatory death

The validity of controlled DCD protocols [11] have been called into question, with opponents focusing on the notion of *irreversibility *incorporated in the Uniform Determination of Death Act of 1981 [8,9]. The Uniform Determination of Death Act defines death as either (1) irreversible cessation of circulatory and respiratory functions or (2) irreversible cessation of all functions of the entire brain, including the brain stem [12]. In uDCD, compliance with the Dead Donor Rule (DDR) is even further compromised by the absence of resuscitation guidelines and the implications of using advanced, highly invasive organ-preservation techniques. The DDR refers to 2 ethical and legal norms governing the practice of organ procurement. The first stipulates that organs can only be taken from dead persons. The second prohibits the killing of patients for or by organ procurement. The DDR prevents the killing of one person for organs that would save the lives of others.

Although the Uniform Determination of Death Act specifically uses the word *irreversible*, the IOM argues that compliance with the DDR in DCD protocols is ensured because "irreversible" (ie, spontaneous life functions cannot be restored by current medical technology) is commonly understood as "permanent" (ie, spontaneous life functions will not be restored because current medical technology will not be used) [5,13]. The IOM follows Bernat's argument that clinical practice commonly refers to the notion of "permanence" rather than to "irreversibility." Permanence "represents an earlier stage of an inevitable process that rapidly and with complete certainty yields irreversibility" [14]. Permanence is considered as the absolute prognosis of irreversibility. Others contend that although the word *permanence *may convey the absolute accuracy of the "prognosis," it certainly is not synonymous to the determination or diagnosis of death [9,15]. Considering that, in the typical (ie, outside the domain of organ procurement) clinical setting, physicians take their time in pronouncing death, it may be less clear how permanence differs from irreversibility; however, in the context of DCD, when the transplant community and professional organizations advocating transplantation are anxious to pronounce death at the earliest possible point, this difference is critical [16-18].

Neither permanence nor irreversibility is an empirical concept and cannot be empirically determined. Both destruction of the brain and the cessation of its functions are directly observable. Permanence and irreversibility are properties about which we can learn only by inference from prior experience. They are not observable conditions. Hence, they cannot serve as evidence, nor can they rightly be made part of an empirical criterion of death.

Destruction of the brain is what convinces us of permanence and irreversibility. But, if there is no proof of complete destruction, then any declaration that a cessation of function is absolutely permanent or irreversible is a presumption, even if well grounded, that is contingent on the current state of medical knowledge and on the availability of adequate life-support systems in the concrete circumstances. Right or wrong, a presumption of permanence or irreversibility of a lack of brain function is insufficient ground for removing a patient's vital organs or for immediate autopsy, cremation, or burial. "To regard the [permanence or] irreversibility of cessation of brain function (at best, a deduction from a set of symptoms) as synonymous or interchangeable with destruction of the entire brain (one but not the only possible cause of these symptoms) is to commit a compound fallacy: identifying the symptoms with their cause and assuming a single cause when several are possible" [19].

**Figure 1:**
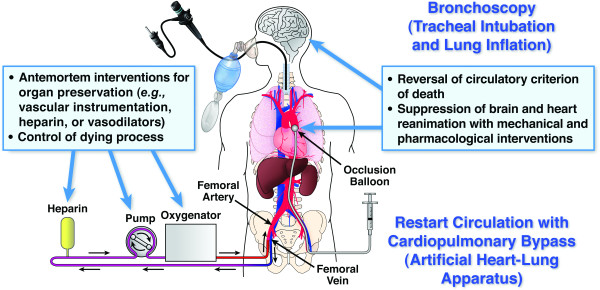
**Organ-donation-related procedures for temporary organ preservation in donation after cardiac death**. The Health Resources and Services Administration of the Department of Health and Human Services and the Organ Procurement and Transplantation Network bylaws include extracorporeal membrane oxygenation with a cardiopulmonary bypass machine (artificial heart-lung apparatus), as well as bronchoscopy, as donation-related procedures for organ preservation in donation after cardiac death [20]. The use of a cardiopulmonary bypass machine (artificial heart-lung apparatus) is initiated for the artificial circulation of oxygenated blood necessary for organ preservation, which reverses the circulatory criterion of death. Tracheal intubation and lung insufflations are required for bronchoscopy (permission to reproduce the figure was obtained from Springer Science and Business Media [21]).

The notion of irreversibility has been further challenged by the decision of the Health Resources and Services Administration of the Department of Health and Human Services and the Organ Procurement and Transplantation Network in 2007 to include extracorporeal membrane oxygenation (ECMO), as well as bronchoscopy, as donation-related procedures in DCD (figure 1) [20]. These donation-related procedures can reverse the circulatory and respiratory criteria used for declaring death and organ procurement [21]. Organ-preservation techniques in uDCD have evolved from the use of cooling blankets, the insertion of catheters in the femoral artery and vein, and the introduction of cooling solution through the arterial catheter into the abdominal cavity to more extensive and invasive procedures. These include the use of automated chest-compression devices, the reinstitution of mechanical ventilation, and the transportation to hospitals for the initiation of normothermic ECMO. Artificial support of circulation with cardiopulmonary bypass and reintubation for lung ventilation, however, can resuscitate these patients during organ procurement. Resuscitated patients who are designated as donors then require pharmacological agents, aortic occlusion of coronary and cerebral circulation, or both to suppress spontaneously returned cardiac activities and neurological functions [22,23]. Studies have shown the effectiveness of ECMO and cardiopulmonary bypass for the return of full neurological functioning after prolonged refractory circulatory arrest [24-26], attesting to the resilience of the human brain to cessation of short periods of circulatory arrest [27,28]. Any claim that criteria in uDCD mirror traditional death, ie, absence of respiration, circulation, and consciousness, appears unsubstantiated. Studies have shown that the use of ECMO in cardiac patients unresponsive to cardiopulmonary resuscitation can result in a 31.6% survival to hospital discharge [29]. Declaring patients dead before initiating ECMO (as in uDCD) can deprive some patients of the chance of survival and full recovery. Therefore, not only is it that no universal guidelines exist that identify the duration until resuscitative efforts are stopped, but also "it is how you get resuscitated that determines if your death is irreversible" [30], although there can never be return to life after true death.

The use of ECMO makes the conclusion even more likely that patients enrolled in DCD protocols are not actually dead at the time of organ procurement which violates the DDR [31-34]. The probability of return of signs of life or the need for suppression of signs of life during surgical procurement would make the act of procurement an act of homicide or mercy killing [16-18]. This medical practice conflicts with how the Center for Medicare and Medicaid Services defines a DCD individual in 42cfr486.302--"an individual who donates after his or her heart has irreversibly stopped beating" [35]. We have previously argued that, if organs are indeed removed from persons without compliance with the legal definition of death outlined in the Uniform Determination of Death Act [36], then enforcement of federal policies and state legislations promoting such an activity can also be construed as a direct violation of the Fourteenth Amendment of the United States Constitution (1868): "No State shall make or enforce any law which shall abridge the privileges or immunities of citizens of the United States; nor shall any State deprive any person of life, liberty, or property, without due process of law; nor deny to any person within its jurisdiction the equal protection of the laws." The Health Resources and Services Administration's endorsement of the use of ECMO is opposite to what the US Congress intended from 42 USC section 1441: to prevent using federal funds encouraging activities such as assisted suicide, euthanasia, and mercy killing [37].

## Presumed authority to initiate TOP techniques

In the context of uDCD, one critical question in a system of expressed consent is how to address the need for organ preservation when a potential donor dies unexpectedly and no proof of recorded expression of donor status is available. Can there be an ethical warrant for undertaking TOP techniques under such conditions? The IOM recommended that community authorization (ie, a switch from expressed to presumed consent) is to be obtained "for the use of postmortem organ preservation techniques during the time needed to seek family consent for donation when the decedent's intention is unknown" [5]. Note that postmortem never can be premortem. To justify presumed consent for TOP, the IOM postulates that there is broad public support for the premise that "a presumption of consent to use preservation techniques enhances rather than limits autonomy by enabling a decision about whether to donate; absent such presumed permission, the opportunity to donate is irretrievably lost" [5]. Since then, some members of the IOM have expressed doubts about the need for language of presumed consent for TOP, arguing instead that current law already allows such interventions. In addition, the IOM describes TOP as a brief, modestly invasive procedure that enhances rather than limits autonomy. This rationale is by itself thought sufficient to justify TOP [13]. Some proponents go one step further embracing the view that it may be even obligatory to preserve the deceased person's organs for a reasonable time while consent for donation from a responsible family member is being sought [38]. In conclusion, the IOM's position rests on the premise that TOP (1) is brief and modestly invasive, (2) enhances autonomy, and (3) is sanctioned by current law. However, the length of time for TOP is nonspecific and depends on how quickly a surrogate decision maker can be located, how fast consent for organ procurement can be obtained, or how long it takes for refusal of donation to be accepted by organ procurement organization (OPO) personnel. Furthermore, placement of any foreign material, such as a temporary catheter into a blood vessel or a body cavity, or infusion of any type of solution or medication into a person's blood stream is considered invasive in everyday medical practice. Finally, although the IOM did not recommend ECMO in its 2006 report, since then, the use of this technology has quickly become mainstream in medical practice and has been recommended in practice guidelines for controlled DCD organ procurement for transplantation [39]. Its use in uDCD protocols, though, remains controversial.

The argument that presumed consent for organ preservation enhances autonomy seems odd, although it effectively obscures the utilitarian objectives of DCD. It is odd because respect for autonomy is the primary reason for having a system of informed consent in medical practice. To argue that autonomy is enhanced by violating it in the first place appears illogical, particularly when utilitarian priorities rather than the best interests of the patient even when he or she is considered to be a potential donor, are disproportionately represented in the consenting process for organ procurement. In other words, a physician presuming consent must intend to promote and materialize the best interests of the patient. In the same manner, a surrogate decision maker must intend to act in accordance with that same objective. Transplant professionals implementing TOP under the assumption of "presumed consent" are eroding the DDR for a hypothetical "opportunity to donate" that benefits others but not the dying patient. The opportunity to donate does not serve the patient qua patient, unless the patient's consent to donation is genuinely and freely expressed (with full decision-making capacity), a condition not normally achieved in a vulnerable "donating" population, where "presumption" rules.

In regard to organ donation, the US has in place a system of expressed consent or opting in. It can be argued that such a system introduces a problem when expressed consent to donation is absent and the risk of losing organs for transplantation must be minimized. In most other circumstances in which expressed consent is legally required, however, ie, legal documents such as wills and contracts, absence of consent entails no consent. Considering the objective to maximize procurement opportunities, it is believed important to reduce the risk of loss of transplantable organs. The legitimate question is then how society should legally deal with absent consent to donate. It is reasonable to assume that at least some who did not provide expressed consent to donate did so because of reasons other than dissent. In those cases, presumed intent to donate and the initiation of TOP would mean that the person's autonomy is being protected, although unknowingly. There are others who intentionally have not consented but did not have a way to document refusal. In these cases, TOP would violate their right to autonomy. It would be equally valid to argue that enhancement of autonomy is better served by assuming that all who did not consent did so with intent. Suggestions that, in the absence of mandatory informed decision making, an opportunity for surrogate informed decision making should be allowed because of presumed uncertainty about whether individuals, prior to the unanticipated condition, may have wanted to change the donation decision, might also undermine the autonomy of those individuals who did not express consent based on a reflective informed decision not to donate. An additional argument against allowing presumed intent is that, in all other situations in life, granting second-opportunity contemplation is not an option and would have serious implications, for instance, for the legal system. Once someone commits a crime, the legal system does not give a person a second chance by arguing that, by being given a second opportunity, the person's autonomy will be enhanced and better decisions might be made. Other commentators have argued that surrogate consent in DCD should not be allowed as consent is unlikely to be based on a best-interest analysis, "even if the adult wanted to be an organ donor, because the incompetent adult's condition will not improve as a result of the interventions" [40] or even the evaluations to declare "brain death" by apnea testing.

Respect for a person's autonomy requires respect for the person's decision not to register as an organ donor, evidenced by the absence of registration information. To presume donation intent, even when a person is not registered as a donor, to allow time to confirm nonregistration or to second guess the autonomous decision of nondonors, is inconsistent with other moral and legal practices in society. If the objective is to enhance individual autonomy, it seems that a system of mandated informed decision making would be a better-suited model. It would require everyone to express their autonomously made informed decision about organ donation and that decisions be recorded in easily accessible registries [8].

## Potential for conflicts of obligation, loyalty, and interests

The disparity between the supply and the demand for transplantable organs has triggered a variety of initiatives to increase the number of organs available for transplantation. In all of these initiatives, the interests of 2 important stakeholders are of concern in DCD, (1) the patients designated as potential or prospective donors and (2) the persons in need of a transplantable organ [13]. It must be recognized that, although both groups should be considered to be primary stakeholders, other agents have critical interests as well. Among those are OPOs, medical institutions, pharmaceutical companies, and individual providers associated with transplantation practice. Financial incentives, institutional and professional prestige, and the belief that the saving of human lives allows for moral boundaries to be pushed out are in play as well [41]. Transplant advocates exploit the term "organ shortage" to gain public support for implementing policies that can have serious sociocultural and legal consequences. The term organ shortage " [seems] to be based on the situation formed from the point of views of demand, that is, of (former, potential or prospective) recipients that there is a demand-driven market for organs, and thus takes side with the perspectives, interests and concerns of only one of the parties involved on organ donation" [42]. This biased approach becomes apparent in the limited information disclosure in public surveys about scientific, ethical and medical concerns regarding death declaration in procurement practices, subsequently perpetuating distorted views of the consequences of a nonconsensual TOP policy for the principal stakeholders, ie, persons designated as potential donors [43]. Regardless of the wide variety of interests, policy making has focused predominantly on facilitating procurement processes and maximizing opportunities for what is still called organ *donation*. Federal regulations require Medicare-approved hospitals and transplant centers to have in place policies and procedures for DCD [44]. In all of the policy activities, the interests of patients who are designated as potential donors and their families appear to have lower priority. As we indicated earlier, others have argued, instead, that contemporary policies strengthen donors' interests by firming up donors' autonomy [5].

The Department of Health and Human Services announced, in 2003, the formation of the Organ Donation Breakthrough Collaborative, which in turn created 58 national donation service areas to organize the transplant community across the United States [45]. The goal of the Organ Donation Breakthrough Collaborative is for hospitals within each donation service area to reach a target cadaveric organ donation rate of 75% or higher. The Uniform Anatomical Gift Act (UAGA) was revised in 2006 in accordance with the controlling federal regulation section 42 CFR §482.45 mandating hospitals to notify the OPOs in each donation service area when a person's death is imminent or has occurred for possible organ and tissue procurement [46]. The revised UAGA sections 14c and 21b create the default rule of initiation and/or continuation of life-support systems for organ preservation until the evaluation of medical suitability of organs for transplantation has been completed and overriding a prospective donor's expression not to have life prolonged by life-support systems unless an expression of contrary intent is documented [47]. Sections 14c and 21b have been justified based on the federal mandate of referring *all *hospital patients at the end of life for possible organ and tissue procurement. The federal mandate is put in place despite the fact that life-support systems are necessary for preserving organs at the end of life and can inflict unwarranted traumatic and distressing experiences to patients seemingly close to death but still living and to their families [48]. The rationale for this decision is clarified in the1987 amendments of the UAGA: encouraging organ donation is the primary purpose [46]. The drafters of the UAGA went so far as to include a good-faith immunity clause to protect medical personnel from civil and criminal liability for efforts they make to comply with the Act. To further promote organ donation and not to frustrate the underlying goal of the Act, physicians, hospital staff, and others are encouraged to read the Act liberally [38]. Under the heading of continuous quality improvement, the use of the Rapid Assessment of Hospital Procurement Barriers in Donation has been proposed [49]. This quality-improvement program measures compliance of the hospital staff with procurement policies and procedures; it recommends implementation of "corrective" interventions to change attitudes and behaviors deemed necessary to improve the rate of organ procurement, and it raises the possibility of future punitive action against hospitals assigned a "poor" rating. The Rapid Assessment of Hospital Procurement Barriers in Donation program aims to mold the psychosocial characteristics of the hospital staffs' knowledge of and adherence to policies regarding donation, patient advocacy, and the hospital-OPO relationship to create a greater compatibility with nationally used strategies that optimize organ procurement.

Federal regulations also mandate that discussing organ donation must be initiated by a certified procurement coordinator, a situation that, in turn, creates a potential conflict between the medical team and procurement coordinators [44]. Priorities and interests may collide when the timing of these conversations with family members is off. To foster a level of trust with family members of potential donors, OPOs, consistent with the Organ Donation Breakthrough Collaborative guidelines, have used a collaborative approach (ie, "team huddle program") linking procurement coordinators with the medical team responsible for patient care at an early stage after hospital admission and before making end-of-life decisions [50,51]. However, team huddling, particularly in circumstances of great emotional distress, can also contribute to increased confusion for families: who is who and whose interests do they represent?

In a civil case (Jacobs v. The Center for Organ Recovery & Education and others) in the United States District Court for the Western District of Pennsylvania, a wrongful death suit was filed by the parents of Gregory Jacobs in March of 2009 [52]. Plaintiffs claim that 18-year-old Gregory was intentionally killed at the hospital so that his organs could be harvested. Gregory sustained a head injury while snowboarding. The suit alleges that he "experienced neither a cessation of cardiac activity nor a cessation of brain activities when surgeons began the procedures for removing his vital organs. But for the intentional trauma or asphyxiation of Gregory Jacobs, he would have lived, or, at the very least, his life would have been prolonged." In addition, the plaintiffs claim that the organ-procurement coordinator and the physicians involved agreed to mislead Gregory's parents into believing that Gregory was "brain dead" and had no chance of survival so that they could harvest his organs. Gregory's father was told that Gregory was "brain dead." The case alleges that the defendants knew that this was false but communicated this to convince Gregory's father to give consent for organ donation. The OPO representations, through the coordinator, according to the complaint, were fraudulent and deceptive, resulting in confusion and misunderstanding, thereby violating the Unfair Trade Practices and Consumer Protection Law. This case illustrates how confusing and potentially deceptive (real or perceived) the interactions with OPO coordinators can be. The concept of team huddling may create only the perception of a trustworthy relationship, which could easily result in the destruction of (public) trust in the long run. The immunity clause in the UAGA may protect against allegations of wrongful death. It is unclear, however, whether persons can be held liable for injuries arising from the determination of death itself [53]. Considering what is outlined above, it seems that the interests of all stakeholders appear to have received high priority, except for the interests of the donor. Physicians caring for critically ill patients may therefore be placed in a position of considerable moral conflict [30]. This moral conflict pertains to patient-care decisions for the potential donor, including decisions to stop resuscitation interventions and end-of-life care, and pressures due to, among others, institutional performance expectations and regulatory compliance.

## Conclusion

The practice of DCD in general is problematic because of the absence of an agreement on criteria for declaring death and guidelines for duration to stop resuscitation efforts and techniques. From a legal point of view, the organ procurement itself could be viewed as the proximate cause of death. Although the revised UAGA of 2006 provides broad immunity to those involved in organ-procurement activities, courts in the United States have yet to provide an opinion on whether persons can be held liable for injuries arising from the determination of death itself. Preserving organs requires the administration of life-support systems such as ECMO that could contribute to the return of signs of life that, in turn, have to be deliberately suppressed pharmacologically. Finally, allowing TOP is inherently a violation of the principle of respect for a person's autonomy, which it claims to enhance.

## Abbreviations

DCD: Donation after cardiac death; uDCD: Uncontrolled Donation after Cardiac Death; DDR: Dead Donor Rule; ECMO: Extracorporeal membrane oxygenation; IOM: Institute of Medicine; OPO: Organ procurement organization; TOP: Temporary organ preservation; UAGA: Uniform Anatomical Gift Act.

## Competing interests

No authors have affiliations or financial involvement with any organization or entity with a direct financial interest in the subject matter or materials discussed in the manuscript. The authors declare that they have no competing interests.

## Authors' contributions

The JLV, MYR, and JLM attest they have made substantial contributions in drafting the manuscript and revising it critically for important intellectual content, that they have given final approval of the version to be published, and that they have participated sufficiently in the work to take public responsibility for appropriate portions of the content. JLV, MYR, and JLM read and approved the final manuscript.

## About the authors

Joseph Verheijde is an associate professor of Biomedical Ethics at College of Medicine, Mayo Clinic and adjunct professor, Bioethics, Policy and Law Program, Center for Biology and Society, Arizona State University. Dr. Verheijde is the program director of the Mayo Clinic/Arizona State University Bioethics Internship Program. Mohamed Rady is a professor of medicine at College of Medicine, Mayo Clinic; consultant in the Department of Critical Care at Mayo Clinic Hospital in Phoenix, Arizona, and adjunct professor, Bioethics, Policy and Law Program, Center for Biology and Society, Arizona State University. Dr. Rady serves on the Ethics Committee at Mayo Clinic in Arizona, the Ethics Committee of the Society of Critical Care Medicine, and the American College of Critical Care Medicine. Joan McGregor is a professor of philosophy at Arizona State University. Dr. McGregor has a special interest in the ethics of organ donation and end-of-life care.
